# *MicroRNA-375* Functions as a Tumor-Suppressor Gene in Gastric Cancer by Targeting Recepteur d’Origine Nantais

**DOI:** 10.3390/ijms17101633

**Published:** 2016-09-27

**Authors:** Sen Lian, Jung Sun Park, Yong Xia, Thi Thinh Nguyen, Young Eun Joo, Kyung Keun Kim, Hark Kyun Kim, Young Do Jung

**Affiliations:** 1 Research Institute of Medical Sciences, Chonnam National University Medical School, 501-190 Gwangju, Korea; senlian9@gmail.com (S.L.); gene-pjs@hanmail.net (J.S.P.); xiayong0728@126.com (Y.X.); thinhnt1984@gmail.com (T.T.N.); yejoo@chonnam.ac.kr (Y.E.J.); kimkk@chonnam.ac.kr (K.K.K.); 2 Biomolecular Function Research Branch Division of Precision Medicine and Cancer Informatics, Division of Precision Medicine and Cancer Informatics, National Cancer Center, 410-769 Goyang, Korea; hkim@ncc.re.kr; 3 Department of Biochemistry, Chonnam National University Medical School, 5 Hakdong, 501-190 Gwangju, Korea

**Keywords:** *microRNA-375*, Recepteur d’Origine Nantais, gastric cancer

## Abstract

Emerging evidence supports a fundamental role for microRNAs (miRNA) in regulating cancer metastasis. Recently, *microRNA-375* (*miR-375*) was reported to be downregulated in many types of cancers, including gastric cancer. Increase in the expression of Recepteur d’Origine Nantais (RON), a receptor tyrosine kinase, has been reported in tumors. However, the function of *miR-375* and RON expression in gastric cancer metastasis has not been sufficiently studied. In silico analysis identified *miR-375* binding sites in the 3′-untranslated regions (3′-UTR) of the RON-encoding gene. Expression of *miR-375* resulted in reduced activity of a luciferase reporter containing the 3′-UTR fragments of RON-encoding mRNA, confirming that *miR-375* directly targets the 3′-UTR of RON mRNA. Moreover, we found that overexpression of *miR-375* inhibited mRNA and protein expression of RON, which was accompanied by the suppression of cell proliferation, migration, and invasion in gastric cancer AGS and MKN-28 cells. Ectopic *miR-375* expression also induced G1 cell cycle arrest through a decrease in the expression of cyclin D1, cyclin D3, and in the phosphorylation of retinoblastoma (Rb). Knockdown of RON by RNAi, similar to *miR-375* overexpression, suppressed tumorigenic properties and induced G1 arrest through a decrease in the expression of cyclin D1, cyclin D3, and in the phosphorylation of Rb. Thus, our study provides evidence that *miR-375* acts as a suppressor of metastasis in gastric cancer by targeting RON, and might represent a new potential therapeutic target for gastric cancer.

## 1. Introduction

Gastric cancer remains one of the most frequent malignant diseases, despite its steady decline in incidence worldwide over the last few decades. Overall, gastric cancer is the second leading cause of cancer mortality, based on recent statistics [1]. Due to local invasion and metastasis, the prognosis of patients with advanced gastric cancer is still disappointing. Thus, it is of the utmost importance to fully elucidate the underlying molecular mechanisms of gastric cancer to develop novel therapeutic strategies.

Recepteur d’Origine Nantais (RON) receptor tyrosine kinase, also known as the macrophage-stimulating protein (MSP) receptor [2], has been implicated in malignancy. RON is initially synthesized as a 180 kDa heterodimeric protein which consists of a 40 kDa extracellular α-chain and a 150 kDa transmembrane β-chain [3]. Studies have reported that over-expression of RON was observed in lung [4], breast [5], colon [6], pancreas [7], ovarian [8], bladder [9], and prostate [10] carcinoma. Recent studies have suggested that RON expression is significantly up-regulated in gastric cancer cell lines [11] and in gastric carcinoma tissues [12]. Further evidence has implied that RON can activate c-Abl to promote cell proliferation [13] and can activate c-Src to mediate cell-cycle progression and the survival of tumor cells [14]. In addition, knockdown of RON markedly reduces tumor metastasis [15] and promotes cancer cell apoptosis [7]. Hence, RON warrants further investigation for its role in gastric cancer.

MicroRNAs (miRNAs) are small (approximately 22 nucleotides) non-coding RNAs that regulate more than half of the genes in human cells [16]. These oligonucleotides typically negatively regulate gene expression by binding within the 3′-untranslated region (3′-UTR) of target messenger RNAs [17,18]. More than half of identified human miRNA genes are located in cancer-associated genomic regions or in fragile sites [19]. miRNAs can be classified as oncogenes or tumor suppressors, and through targeting various transcripts they participate in a diverse range of processes including cellular differentiation, proliferation, metabolism, and death [20,21,22]. Microarray analysis of miRNA expression has revealed that a number of miRNAs are aberrantly expressed in carcinoma tissues [23,24]. Recently, aberrant expression of miRNA has frequently been reported in gastric cancer, suggesting that miRNAs are deeply involved in metastasis in this type of cancer [25,26,27,28].

Accumulating evidence has revealed that *miR-375* is widely expressed in various tissues and organs and is significantly reduced in malignant cells, for instance, hepatocellular carcinoma [29], breast cancer [30], esophageal carcinoma [31], and head and neck cancer [32]. Furthermore, miR-375 has been identified as a tumor-suppressor in gastric cancer by targeting PDK1, 14-3-ζ [33], and JAK2 [34]. *miR-375* has also been shown to inhibit YAP1 expression [35], the transcription factor SP1 [36], and the wnt/β-catenin pathway [37]. However, to the best of our knowledge, the functions and mechanisms of *miR-375* in regulating RON expression in gastric cancer have not been reported until now.

## 2. Results

### 2.1. miR-375 Directly Targets the 3′-Untranslated Region (3′-UTR) of Recepteur d’Origine Nantais (RON)

**Figure 1:**
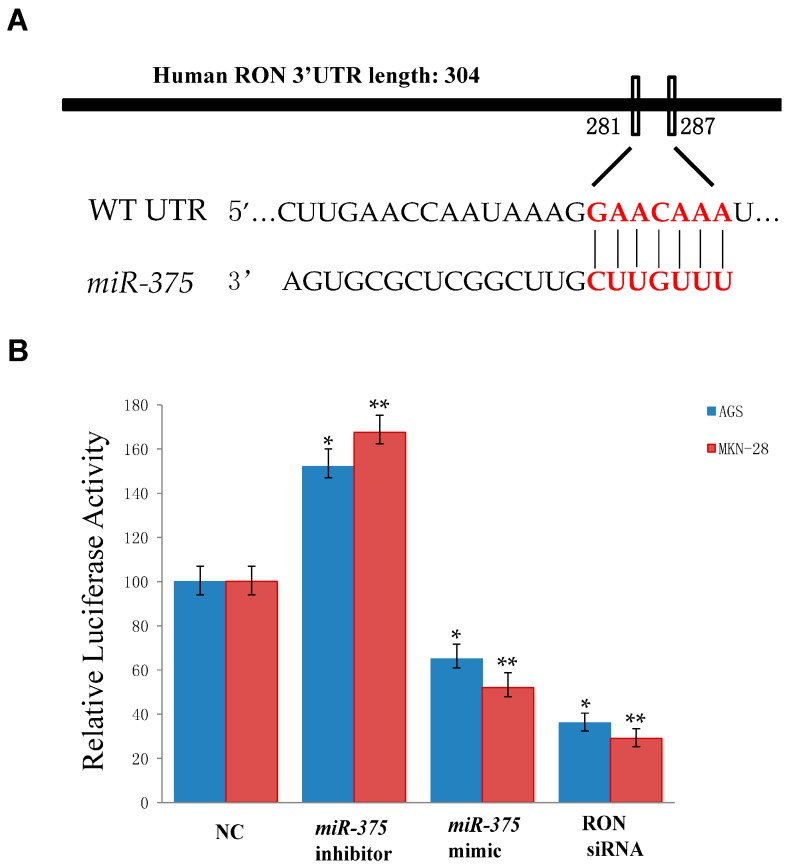
Recepteur d’Origine Nantais (RON) is a *miR-375* target. (**A**) Schematic representation of the *miR-375*-binding sites in the RON Recepteur d’Origine Nantais (3′-UTR) region, as detected by TargetScan; The letters in red color indicates the corresponding potential targets sites of miR-375 in 3′-UTR of RON; (**B**) Luciferase assay in AGS and MKN cells. AGS and MKN-28 cells were co-transfected with a *miR-375* mimic or negative control or RON siRNA and a vector containing the 3′-UTR of RON cloned downstream of a luciferase gene. Relative repression of the luciferase expression was standardized to the β-gal signal. * *p* < 0.05 versus negative control (AGS cell); ** *p* < 0.05 versus negative control (MKN-28 cell); The data represent the mean ± SD from triplicate measurements. NC—negative control.

To elucidate the antitumor effect of *miR-375* in gastric cancer, we employed the TargetStan Release 5.1 online software [38] and PicTar [39] algorithms to search for putative human protein-coding gene targets of *miR-375*. Through prediction in the bioinformatics database, we identified the potential binding capability of *miR-375* to RON 3′-UTR. (Figure 1A). Next, we determined whether *miR-375* directly binds these predicted sites in the 3′-UTR of RON to control expression. AGS and MKN-28 cells were co-transfected with *miR-375* mimic, *miR-375* inhibitor, negative control, or RON siRNA and a reporter expression vector containing the 3′-UTR of RON cloned downstream of a luciferase gene. The luciferase activity in cells co-transfected with the *miR-375* mimic was significantly decreased compared to that in cells co-transfected with the negative control (Figure 1B). Moreover, the luciferase activity in cells co-transfected with RON siRNA was suppressed compared to that in cells co-transfected with the negative control (Figure 1B). Thus, these results indicated that RON was a direct target of *miR-375*.

### 2.2. Inverse Association between miR-375 and RON Expression in AGS and MKN-28 Human Gastric Cancer Cells

**Figure 2:**
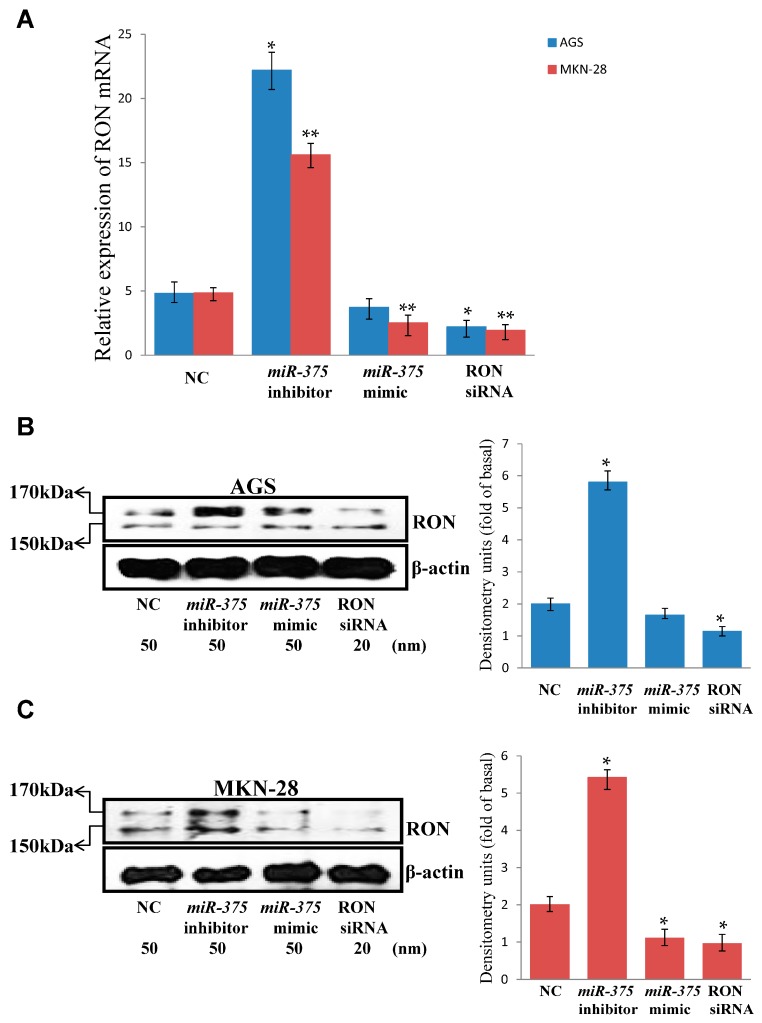
Inverse relationship between the expression of *miR-375* and RON in AGS and MKN-28 cells. (**A**) RON mRNA levels after transfection of AGS and MKN-28 cells with a *miR-375* mimic, a *miR-375* inhibitor, a negative control, or RON siRNA. Total RNA was extracted 48 h after transfection and mRNA levels were determined by qRT-PCR. * *p* < 0.05 versus negative control (AGS cell); ** *p* < 0.05 versus negative control (MKN-28 cell); The data represent the mean ± SD from triplicate measurements; (**B**) RON protein levels after transfection of AGS cells with a *miR-375* mimic, a *miR-375* inhibitor, a negative control, or RON siRNA. Total cell lysates were extracted 48 h after transfection and protein levels were determined by Western blot. * *p* < 0.05 versus negative control; The data represent the mean ± SD from triplicate measurements; (**C**) RON protein levels after transfection of MKN-28 cells with a *miR-375* mimic, a *miR-375* inhibitor, a negative control, or RON siRNA. Total cell lysates were extracted 48 h after transfection and protein levels were determined by Western blot. * *p* < 0.05 versus negative control; The data represent the mean ± SD from triplicate measurements.

To assess further that RON is a target of *miR-375*, we examined RON mRNA and protein levels in *miR-375* mimic-transfected, *miR-375* inhibitor-transfected, and negative controls by quantitative reverse transcription-PCR (qRT-PCR) and Western blotting. Overexpression of RON mRNA (Figure 2A) and RON protein (Figure 2B,C) was observed in *miR-375*-inhibitor-transfected cells, when compared to those of the negative control. The expression of RON at the mRNA (Figure 2A) and protein level (Figure 2C) was reduced after forced expression of *miR-375*, compared to those of the negative control in MKN-28 cells. However, the expression of RON at the mRNA (Figure 2A) and protein level (Figure 2B) was not obviously reduced after forced expression of *miR-375*, compared to those of the negative control in AGS cells. Furthermore, we observed a clear reduction in the levels of RON mRNA and protein in RON siRNA-transfected cells compared to those in negative control-transfected cells (Figure 2A–C). Taken together, our results demonstrate an inverse association between *miR-375* and RON in AGS and MKN-28 human gastric cancer cells.

### 2.3. miR-375 Inhibits AGS and MKN-28 Human Gastric Cancer Cell Proliferation

**Figure 3:**
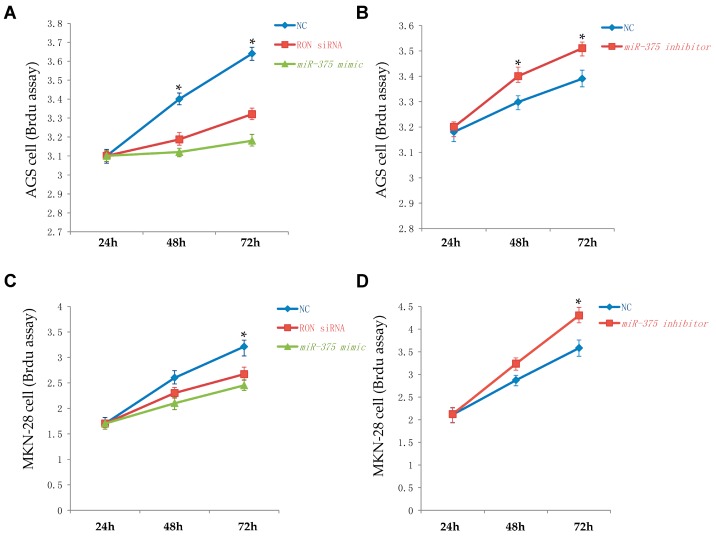
*miR-375* and RON regulate cell proliferation in AGS and MKN-28 human gastric cancer cells. (**A**) Brdu assay in AGS cells transfected with a miR-375 mimic, a negative control, or RON siRNA; (**B**) Brdu assay in AGS cells transfected with a miR-375 inhibitor or a negative control; (**C**) Brdu assay in MKN-28 cells transfected with a miR-375 mimic, a negative control, or RON siRNA; (**D**) Brdu assay in MKN-28 cells transfected with a miR-375 inhibitor or negative control. * *p* < 0.05; The data represent the mean ± SD from triplicate measurements.

Cell proliferation was evaluated using a cell proliferation ELISA Brdu Colorimetric kit based on a Brdu (bromodeoxyuridine) incorporation assay. AGS and MKN-28 cells were transiently transfected with a *miR-375* mimic, a *miR-375* inhibitor, or a negative control. The proliferation rate of AGS and MKN-28 cells at 72 h after transfection with the *miR-375* mimic was significantly reduced compared to that of the miRNA negative control (Figure 3A,C). The proliferation rate of AGS and MKN-28 cells after transfection with the *miR-375* inhibitor was slightly increased compared to that with the miRNA negative control (Figure 3B,D). These results suggest that overexpression of *miR-375*, in AGS and MKN-28 cells, inhibits cell proliferation.

### 2.4. miR-375 Induced G1 Cell Cycle Arrest by Decreasing Cyclin D1 and Cyclin D3 Expression, and Retinoblastoma (Rb) Phosphorylation

**Figure 4:**
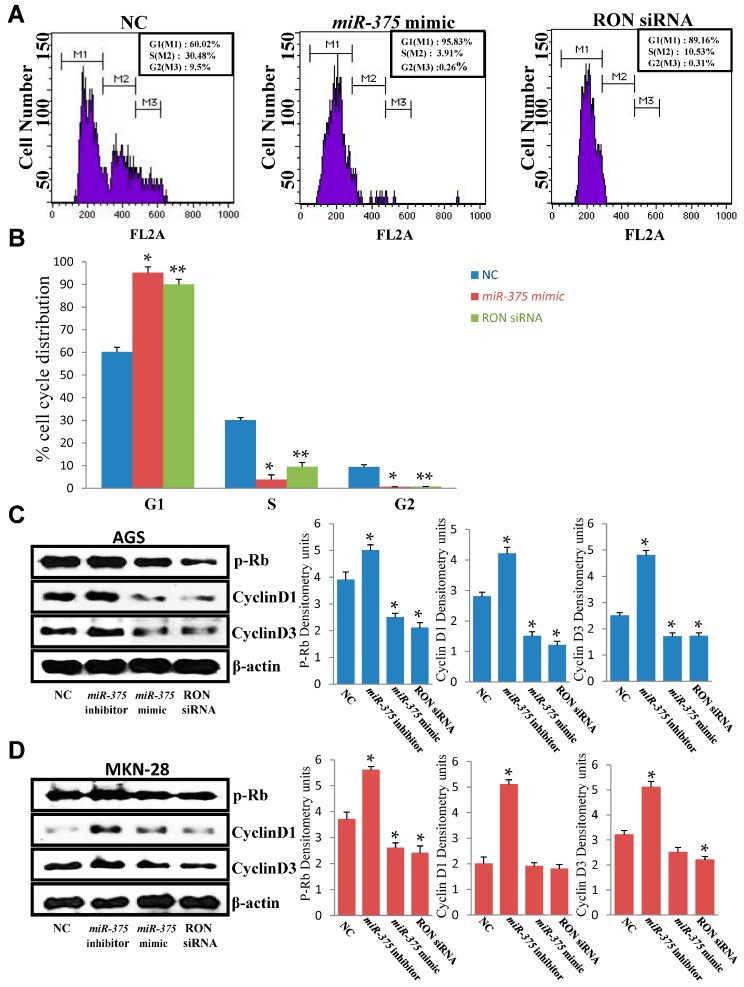
*miR-375* and RON regulate the cell cycle by decreasing cyclin D1 and cyclin D3 expression and retinoblastoma (Rb) phosphorylation in AGS and MKN-28 human gastric cancer cells. (**A**,**B**) Representative histograms show cell cycle distribution of AGS cells in the negative control, *miR-375* mimic, and RON siRNA groups. * *p* < 0.05 versus negative control (*miR-375* mimic); ** *p* < 0.05 versus negative control (RON siRNA); (**C**) Cyclin D1, cyclin D3, and phosphorylation of Rb were assessed by Western blot analysis 48 h after transfection with a *miR-375* mimic, a *miR-375* inhibitor, a negative control, or RON siRNA in AGS cells. * *p* < 0.05 versus negative control; The data represent the mean ± SD from triplicate measurements; (**D**) Cyclin D1, cyclin D3, and phosphorylation of Rb were assessed by Western blot analysis 48 h after transfection with a *miR-375* mimic, a *miR-375* inhibitor, a negative control, or RON siRNA in MKN-28 cells. * *p* < 0.05 versus negative control; The data represent the mean ± SD from triplicate measurements.

The cell cycle in AGS cells was analyzed by flow cytometry 48 h after transfection with a negative control, a *miR-375* mimic, or RON siRNA. Results showed that the percentage of cells in the G1 phase was significantly increased, and the percentage in the S phase was significantly decreased in AGS cells transfected with the *miR-375* mimic or RON siRNA, compared to those in the negative control group (Figure 4A,B). Cyclin-D forms a complex with and functions as a regulatory subunit of CDK, which is required for the cell cycle G1/S transition [40]. Consistently, our results showed downregulation of cyclin D1, cyclin D3, and phosphorylated Rb in *miR-375* expressing AGS cells (Figure 4C). However, the expression of cyclin D1 and cyclin D3 was not significantly reduced after the forced expression of *miR-375* (Figure 4D). These results indicated that *miR-375* regulated G1 cell cycle phase arrest through inhibition of cyclin D1 and cyclin D3, phosphorylation of Rb in AGS cells, and through the inhibition of phosphorylation of Rb in MKN-28 cells.

### 2.5. miR-375 Inhibits Migration in AGS and MKN-28 Human Gastric Cancer Cells

**Figure 5:**
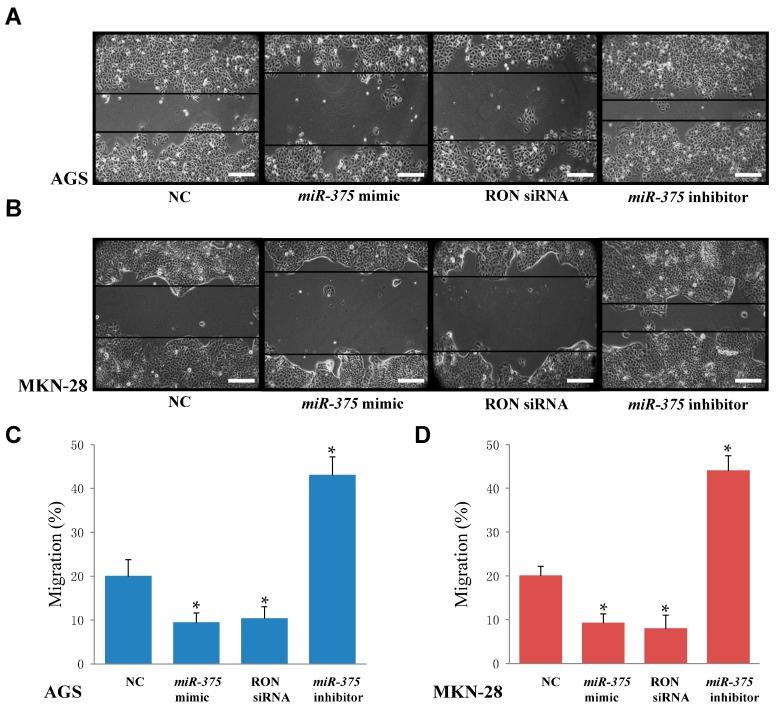
Overexpression of *miR-375* or knockdown of RON inhibits cell migration in AGS and MKN-28 cells. (**A**,**C**) Wound healing assays were performed to evaluate the effect of *miR-375* or RON on the migratory ability of AGS cells; (**B**,**D**) Wound healing assays were performed to evaluate the effect of *miR-375* or RON on the migratory ability of MKN-28 cells. * *p* < 0.05 versus negative control; The data represent the mean ± SD from triplicate measurements. Scale bars: 100 μM in (**A**,**B**). The space between back lines in (**A**,**B**) were measured for the migratory ability of cells.

A wound-healing assay was performed to test the effects of *miR-375* on the migratory ability of AGS and MKN-28 cells. Data indicated that overexpressing *miR-375* (through transient transfection of a *miR-375* mimic) displayed slower wound closure compared to negative controls in AGS cells (Figure 5A,C) and MKN-28 cells (Figure 5B,D). Moreover, cells transfected with the *miR-375* inhibitor showed increased migration compared to the negative control-transfected cells (Figure 5A,B). These findings demonstrated that *miR-375* inhibits the migration of AGS and MKN-28 human gastric cancer cells.

### 2.6. miR-375 Inhibits Invasion in AGS and MKN-28 Human Gastric Cancer Cells

**Figure 6:**
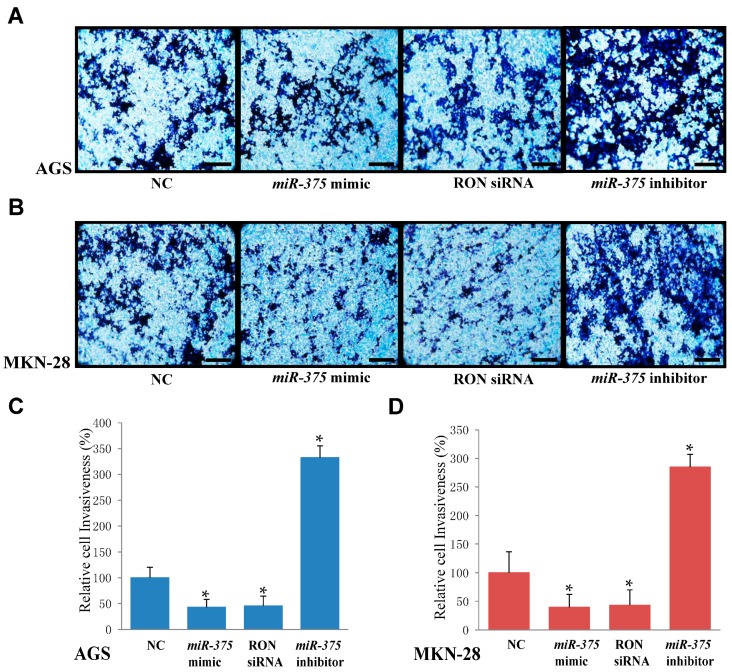
Overexpression of *miR-375* or knockdown of RON inhibits cell invasion in AGS and MKN-28 cells. (**A**,**C**) A modified Boyden chamber assay was performed to evaluate the effect of *miR-375* or RON on the invasive ability of AGS; (**B**,**D**) A modified Boyden chamber assay was performed to evaluate the effect of miR-375 or RON on the invasive ability of MKN-28 cells. * *p* < 0.05 versus negative control; The data represent the mean ± SD from triplicate measurements. Scale bars: 100 μM in (**A**,**B**).

To examine the effect of *miR-375* on tumor invasion, we utilized a modified Boyden invasion chamber. This assay revealed that the invasive capacity of AGS (Figure 6A,C) and MKN-28 cells (Figure 6B,D) was significantly suppressed in cells overexpressing *miR-375* compared to that in the negative control group. In addition, invasion was dramatically enhanced in AGS and MKN-28 cells transfected with a *miR-375* inhibitor compared to that in the negative control cells (Figure 6A,B). These results provided evidence that *miR-375* inhibits the invasiveness of AGS and MKN-28 cells.

### 2.7. Knockdown of RON Reduces Proliferation, Migration, and Invasion in AGS and MKN-28 Human Gastric Cancer Cells

Previous studies suggested that the expression of RON plays a pivotal role in cell proliferation [41], cell migration [42], and cell invasion [43] in several kinds of cancer cells. To determine the role of RON in proliferation, migration, and invasion in human gastric cancer cells, RON was targeted with siRNA. Our results suggested that the knockdown of RON inhibited cell proliferation (Figure 3A,C), cell migration (Figure 5A,B), and cell invasion (Figure 6A,B) in AGS and MKN-28 cells. Song et al. previously reported that RON is associated with cell cycle arrest in gastric cancer cells [44]. In this study, knockdown of RON also markedly induced G1 arrest in AGS cells (Figure 4B). In summary, our results suggest that overexpression of RON causes proliferation, cell cycle arrest, migration, and invasion in human gastric cancer cells, and that *miR-375* serves as a tumor suppressor by targeting RON in human gastric cancer cells.

## 3. Discussion

Tumor metastasis is the leading cause of cancer-related mortality, and is classified as the degradation of the cellular basement membrane and the spread of cancer cells to distant organs, resulting in the formation of secondary tumors. Anti-cancer therapy for gastric cancer remains a major clinical challenge. miRNAs have been identified as important regulators of tumor metastasis, through the regulation of various molecular pathways [19]. These molecules negatively regulate target proteins through suppressing mRNA translation and/or enhancing mRNA degradation [45]. Previous studies have shown that miRNAs are involved in cell differentiation, proliferation, migration, and apoptosis [46,47,48]. Recently, *miR-375* was reported to be associated with cancer development and was shown to be downregulated in head and neck [32] and hepatocellular [29] carcinomas. Importantly, Tsukamoto et al. demonstrated that *miR-375* is the most downregulated miRNA in gastric carcinomas [33]. In the present study, we found that the overexpression of *miR-375* in AGS and MKN-28 gastric cancer cells decreased cell proliferation, migration, and invasion, and induced G1 cell cycle arrest. The observations that ectopic expression of *miR-375* inhibited tumor metastasis is in line with previous findings on squamous cervical cancer cells [36]. Therefore, we report the evidence that *miR-375*, as a tumor suppressor, plays an important role in the progression and metastasis of gastric cancer. 

RON, a member of the c-Met family of scatter factor receptors, has been shown to play an important role in the development, progression, and metastasis of gastric carcinoma [49]. The transcription factors, early growth response-1 and nuclear factor-κB, have been reported to be critical for the expression of RON [43]. Here, we used a public algorithm and found that RON is a putative target gene for *miR-375*, mediating cell proliferation, migration, and invasion. We confirmed the existence of a binding site for *miR-375* in the RON 3′-UTR through a luciferase activity assay. Furthermore, RON mRNA and protein expression levels were significantly decreased by the overexpression of *miR-375* using a *miR-375* mimic. We also demonstrated that knockdown of RON by siRNA inhibited tumor metastasis-related properties in gastric cancer cells, similar to that observed for *miR-375* overexpression. In summary, we have shown that RON is a direct target of *miR-375*, and is related to the development of gastric cancer. In addition, both *miR-375* and RON might represent novel therapeutic targets, as either restoring *miR-375* expression or blocking RON expression could suppress malignant cell behaviors in gastric cancer cells. Further investigations are necessary to address the correlation between *miR-375* and RON in clinical samples. It is worth noting that the overexpression of *miR-375* partially inhibits RON expression. Therefore, we must consider that other mechanisms might also participate in the reduction of RON expression. These studies merit further investigation in the future. 

Cell cycle defects are frequently mediated by misregulation of CDK activity, which is regulated by the binding of positive effectors, specifically cyclins, to negative regulators such as cyclin-dependent kinase inhibitor (CDKI) [50]. Several studies have demonstrated that upregulation of CDKs and dysregulation of CDK inhibitors lead to tumor progression [51]. It has been shown that Rb is a major G1 checkpoint that blocks S-phase entry and cell growth [52]. In this study, we evaluated the effect of *miR-375* on the expression of cyclin D1, cyclin D3, and the phosphorylation of Rb. Our results indicated that the overexpression of *miR-375* inhibited the expression of cyclin D1 and cyclin D3 and the phosphorylation of Rb in AGS and MKN-28 human gastric cancer cells. We also found that the knockdown of RON inhibited cyclin D1 and cyclin D3 expression and the phosphorylation of Rb. Our results suggest that *miR-375* can induce G1 cell cycle arrest by inhibiting cyclin D1, cyclin D3, and the phosphorylation of Rb in AGS and MKN-28 gastric cancer cells. Hereby, we conclude that *miR-375* can induce G1 arrest of gastric cancer cells through this mechanism. However, in our experiments, we did not determine the effect of *miR-375* on the expression of CDK inhibitors.

In summary, our results demonstrated that *miR-375* functions as a tumor suppressor in AGS and MKN-28 human gastric cancer cells through the regulation of RON. Our study provides in vitro experimental evidence that overexpression of *miR-375* inhibits malignant properties in gastric cancer cells. It would be beneficial to provide the survival results of *miR-375* and RON in their own cohort in future studies. In conclusion, our findings suggest that effective therapeutic strategies could be developed through the modulation of *miR-375* and its target gene *RON* in gastric cancer.

## 4. Experimental Section

### 4.1. Reagents

OPTI-modified Eagle’s medium, RPMI-1640 medium, fetal bovine serum (FBS), and penicillin-streptomycin were purchased from HyClone (Logan, UT, USA). TrypLE™ Express was purchased from Gibco (Grand Island, NY, USA). Antibodies against cyclin D1, cyclin D3, and phos-Rb were purchased from Cell Signaling Technology (Danvers, MA, USA). An antibody against RON was purchased from Santa Cruz Biotechnology (Santa Cruz, CA, USA).

### 4.2. Cell Culture

The human gastric cancer AGS and MKN-28 cell lines were purchased from the American Type Culture Collection (Manassas, VA, USA). Cells were cultured in RPMI-1640 medium supplemented with 10% FBS and 0.6% penicillin-streptomycin solution and incubated at 37 °C in a 5% CO_2_ humidified atmosphere. 

### 4.3. Cell Transfection

Cells were transfected with Dharmacon miRIDIAN *miR-375* mimic (*miR-375*) or miRIDIAN micro RNA mimic negative control 1 (negative control) (Thermo Fisher Scientific, Lafayette, CO, USA) according to the manufacturer’s instructions. Cells were transiently transfected with a small interfering RNA targeting RON (RON siRNA) (sc-36434; Santa Cruz, CA, USA) according to the manufacturer’s instructions. siRNA and miRNA mimics were used at a final concentration of 30 nm, unless otherwise mentioned.

### 4.4. Luciferase Activity Assay

Construction of the RON promoter reporter was performed as described previously [49]. AGS and MKN-28 cells (5 × 10^5^) were seeded in 24-well plates 24 h before transfection. Co-transfection of miRNA mimics and reporter vectors was performed with Lipofectamine 2000 from Invitrogen (Carlsbad, CA, USA). Luciferase activities were measured at 48 h after transfection using a Dual-Glo luciferase assay system (Promega, Madison, WI, USA). The firefly luciferase activities were normalized to Renilla luciferase activities, and the relative amount of luciferase activity in untreated cells was designated as 1.

### 4.5. RNA Extraction and Quantitative Real Time PCR (qRT-PCR)

Total RNA was extracted with TRIzol reagent obtained from Invitrogen (Carlsbad, CA, USA). One microgram of total RNA was used for the synthesis of first-strand cDNA by using random primers and M-MLV transcriptase (Promega, Madison, WI, USA). cDNA was subjected to PCR amplification with the primer sets for RON and GAPDH using a PCR master mix solution (iNtRON, Seongnam, Gyeonggi-do, Korea). The following primers were used in this study: RON forward, 5′-ACGGCTTAGCGCCACTGAGC-3′ and RON reverse, 5′-CATGTGTGCCACTGTGACGT-3′ (550 bp amplicon), GAPDH forward, 5′-TTGTTGCCATCAATGACCCC-3′, and GAPDH reverse, 5′-TGACAAAGTGGTCGTTGAGG-3′ (836 bp amplicon). The PCR conditions were as follows: pre-denaturation at 95 °C for 30 s, followed by 40 cycles of 95 °C for 5 s, 55 °C for 15 s, and 72 °C for 15 s. PCR product formation was monitored continuously during the reaction using Sequence Detection System software, version 1.7 (Applied Biosystems, Foster City, CA, USA). The final accumulated PCR products were detected directly by monitoring the increase of the reporter dye (SYBR^®^). The expression of RON mRNA in the treated cells was compared to that in control cells at each time point using the comparative cycle threshold (*C*_t_)-method [53]. The quantity of each transcript was calculated according to the instrument manual and normalized to the amount of glyceraldehyde-3-phosphate dehydrogenase (GAPDH), a housekeeping gene.

### 4.6. Preparation of Cell Extracts and Western Blot Analysis

After each experiment, cells were harvested and washed twice with cold PBS. The harvested cells were then lysed in 100 μL of protein extraction solution (iNtRON, Seongnam, Gyeonggi-do, Korea). Cell homogenates were centrifuged at 10,000× *g* for 20 min at 4 °C. Equal amounts of protein (50 μg) were analyzed by Western blot as described previously [54]. Finally, protein bands were visualized by an enhanced chemiluminescence detection kit (Millipore, Billerica, MA, USA) and were scanned by a luminescence image analyzer (Vilber Loumat, France).

### 4.7. Cell Cycle Analysis

The cells were seeded in 6-well plates for cell cycle analysis. At 72 h after transfection, cells were harvested by trypsinization and fixed in 70% ethanol at 4 °C overnight. After washing and resuspending in 100 μL of phosphate-buffered saline, fixed cells were treated with 50 μL RNase (50 mg/mL) at 37 °C for 30 min and stained with 200 μL propidium iodide at 4 °C for 30 min. FACS analysis was performed using a BD FACS Caliber flow cytometer with CELL Quest software (BD Biosciences, San Jose, CA, USA).

### 4.8. Cell Proliferation Assay

AGS and MKN-28 cells were sub-cultured in 96-well plates (approximately 5 × 10^3^/well) overnight and then transfected with the negative control, miR-375, miR-375 inhibitor, or RON siRNA. Cell proliferation was determined at 24, 48, and 72 h after transfection using a cell proliferation ELISA Brdu Colorimetric kit (Roche Diagnostics, Mannheim, Germany), which is based on a Brdu (bromodeoxyuridine) incorporation assay. Cellular viability was quantified colorimetrically, as the absorbance at 370 nm (for the Brdu incorporation assay) using a spectrophotometer (Epoch Biotek, Winooski, VT, USA) at 24, 48, and 72 h after transfection.

### 4.9. In Vitro Wound-Healing Assay

An in vitro wound-healing assay was used to assess tumor cell motility. Briefly, AGS cells (5 × 10^5^/well) and MKN-28 cells (5 × 10^5^/well) were seeded in 12-well plates, cultured overnight, and transfected with the negative control, *miR-375*, *miR-375* inhibitor, or RON siRNA. When cell confluency approached 90%, the cell monolayer was scratched with a sterile plastic tip to create a wound; the cells were immediately washed with growth medium twice to remove floating cells, and then subsequently cultured in RPMI-1640 medium (including 1% fetal bovine serum) at 37 °C in a 5% CO_2_ humidified atmosphere for up to 24 h. At specific time points, images of the plates were acquired using a microscope. Data were summarized based on three assays for each experiment.

### 4.10. Matrigel Invasion Assay

Cell invasion assays were performed using a 10-well chemotaxis chamber (Neuro Probe, Gaithersburg, MD, USA) with an 8-μM pore membrane (Neuro Probe) with RPMI-1640 containing 10% FBS as the chemoattractant in the lower chamber. AGS or MKN-28 cells (10^5^), transfected with the negative control, *miR-375*, *miR-375* inhibitor, or RON siRNA and in a total volume of 300 μL, were added to each chamber. After 24 h, cells that had invaded the Matrigel and 8-μM pore membrane were fixed and stained with the Quick-Diff stain kit (Becton-Dickinson, Franklin Lakes, NJ, USA). After two washes with water, the chambers were air-dried. The number of invading cells were counted using a phase-contrast microscope. Each experiment was performed in triplicate.

### 4.11. Statistical Analysis

All data are expressed as mean ± standard deviation (SD). Statistical analysis was performed with Student’s *t*-test to compare data between two groups. *p*-values < 0.05 were considered to be statistically significant.
